# Caecal volvulus associated with complex small bowel malrotation and congenital bands: A case report and literature review

**DOI:** 10.1093/jscr/rjab040

**Published:** 2021-04-13

**Authors:** Hussam S Khougali, Yousif Hamad, Ahmed Abdelhamid, Valerio DiNicola

**Affiliations:** Department of General Surgery, Worthing Hospital, Worthing, UK; Department of General Surgery, Worthing Hospital, Worthing, UK; Department of General Surgery, Worthing Hospital, Worthing, UK; Department of General Surgery, Worthing Hospital, Worthing, UK

## Abstract

Caecal volvulus is an uncommon condition and represents only 10% of all colonic volvulus. The mechanism is due to hypermobile cecum twisting around it is own mesentery. A 40-year-old female who was previously fit and well-attended hospital with 2 days history of intermittent abdominal pain, mild distention and constipation. X-rays showed twisted and dilated loops of bowel with right hepato-diaphragmatic bowel loops entrapment. A computed tomography scan was the only confirmatory test of caecal volvulus with intermittent bowel symptoms. Emergency laparotomy revealed complex intraoperative bowel loops malrotation with congenital bands and abnormal position of Duodeno-Jejenal flexure. Open right hemicolectomy was the operation of choice for this complex case. Caecal volvulus must be considered as one of the important differential diagnosis of acute abdomen during initial assessment of patients with intermittent bowel symptoms in emergency department in order to avoid any therapeutic delay.

## INTRODUCTION

Caecal volvulus is a rare cause of large bowel obstruction [1]. caecal volvulus accounts for ~1% of intestinal obstructions and 10–40% of cases of colonic volvulus and considers the second most common type of colonic volvulus after sigmoid volvulus [2]. Abnormally, mobile cecum, ileum and ascending colon are tilting and rotating around mesenteric pedicle [2, 3]. A review of the literature revealed 14 reports of volvulus with bowel malrotation occurring without a clear cause. Of the 14 prior reports of such condition, 8 reports were adults and 6 were children. Generally, risk factors include previous abdominal surgeries, intra-abdominal tumors, congenital peritoneal bands, pregnancy, Overexertion and blunt trauma [4, 5].

Patients who are symptomatic often present either acutely with bowel obstruction and intestinal ischemia or chronically with vague abdominal pain that often makes the diagnosis very difficult. These symptoms are caused by peritoneal bands run from the cecum to the right lateral abdominal wall [6]. If no bowel obstruction or compression of bowel blood supply is present, this condition may remain clinically silent, requiring a high index of suspicion in order to avoid any diagnostic delay that may ultimately lead to death [7, 8].

Radiography of the abdomen usually shows characteristic findings include a dilated and displaced haustral bowel loop on the right side of the abdomen, computed tomography (CT) remains the confirmatory study when distended cecum and associated “whirl sign” of twisted mesenteric vessels appear in the scan [9]. In this paper, we review the literature for this rare condition and report on a case of complex type of caecal volvulus.

## CASE REPORT

A 40-year-old lady presented to the emergency department complaining of intermittent abdominal pain, distention and absolute constipation for 2 days with no history of fever, previous abdominal surgery or blunt trauma. She has been previously fit and well with good performance.

On examination, her abdomen was soft, moderately distended and slightly tender over the right upper quadrant region, per rectal examination revealed loose stool in rectum. Her observations and blood investigations were completely unremarkable (Tables 1 and 2).

**Table 1:** Vital signs at the time of presentation to A + E

Pulse	78 p/m
Blood pressure	116/78
Temperature	37 C
Pain score	4/10
Respiratory rate	18
O2 saturation	99%
News score	0

**Table 2:** Bloods investigation

Hemoglobin	109	NR: 120–150 g/L
White cells count	11.5	NR: 4–10 × 10*9/L
Platelets	215	NR: 150–410 × 10*9/L
Neutrophils	8.8	NR: 2.0–7.0 × 10*9/L
Sodium	132	NR: 133–146 mmol/L
Potassium	4.5	NR: 3.5–5.3 mmol/L
eGFR	>90	NR: 60–120 ml/min
CRP	<1	NR: 0.0–5.0 mg/L

NR, Normal range

**Figure 1:**
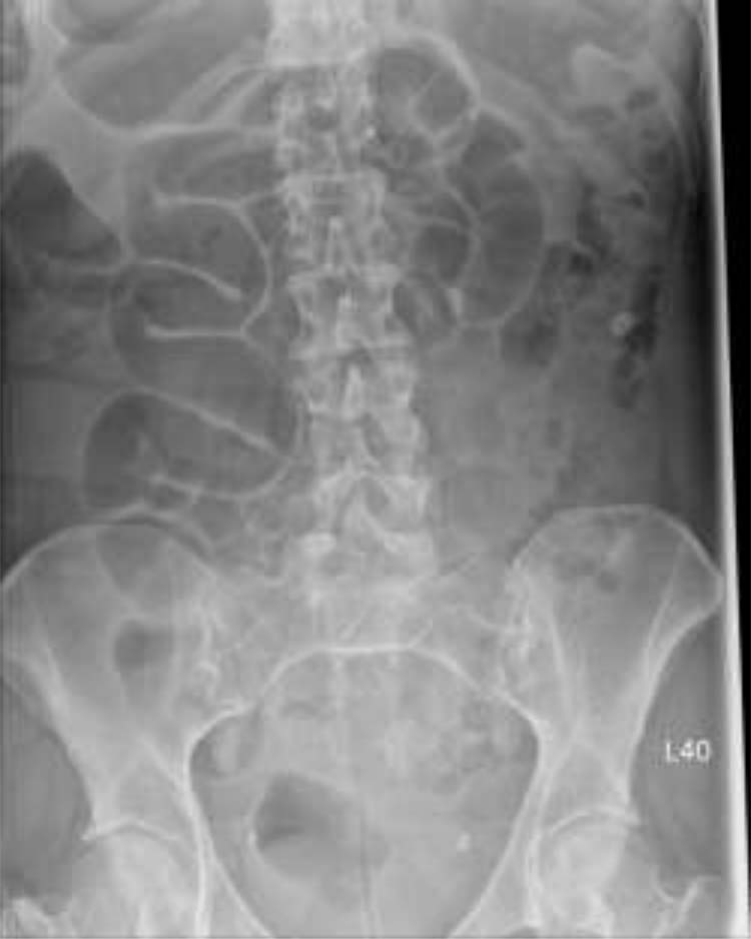
Abdominal X-ray shows twisted dilated small bowel loops.

**Figure 2:**
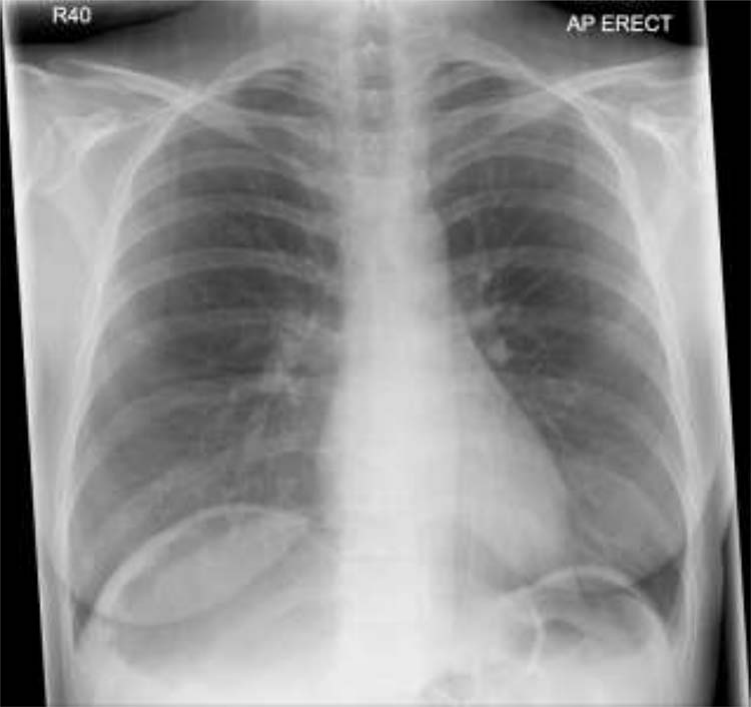
Chest X-ray shows air under diaphragm due to Chilaiditi syndrome.

**Figure 3:**
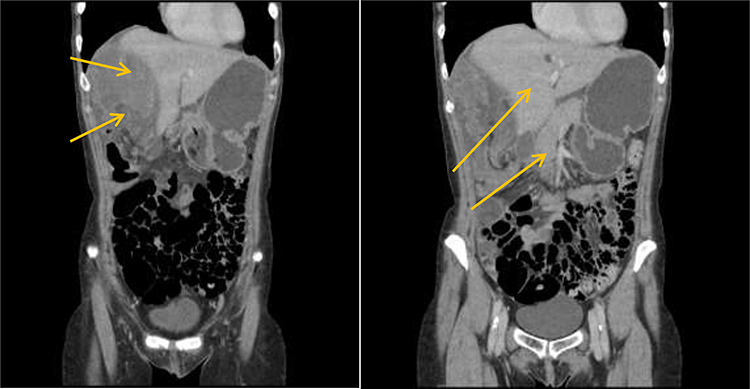
CT scan shows interposition of ileum, cecum and ascending colon between liver and diaphragm pushing right loop of the liver and gallbladder medially.

**Figure 4:**
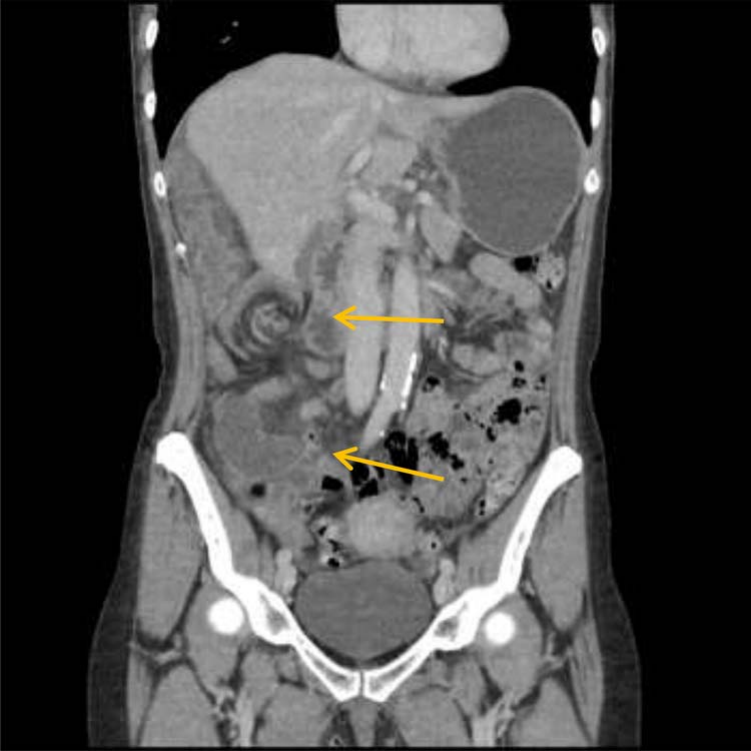
CT scan shows a vascular/mesenteric pedicle swirl suggestive of malrotation or volvulus with evidence of dilated closed loop obstruction.

Patient initially diagnosed as biliary colic and surgical team had been contacted. Plain Abdominal radiograph showed dilated small bowels loops (Fig. 1). Chest radiograph revealed air under right hemi-diaphragm (Fig. 2). CT scan was done in emergency department and confirmed interposition of bowel between the diaphragm and the liver with a vascular/mesenteric pedicle swirl suggestive of malrotation or volvulus of the right colon. There was marked mural thickening and bold dilatation with high attenuation mucosa in keeping with closed loop obstruction and possible necrosis (Figs 3 and 4).

**Figure 5:**
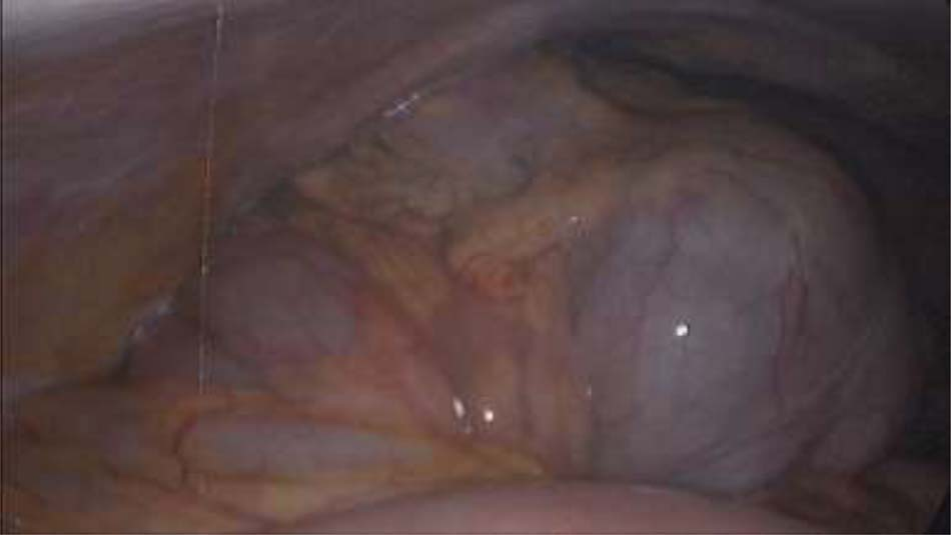
Showing 180 degree volvulus of terminal ileum, cecum and ascending colon overlapped each other and overriding the right loop of the liver.

**Figure 6:**
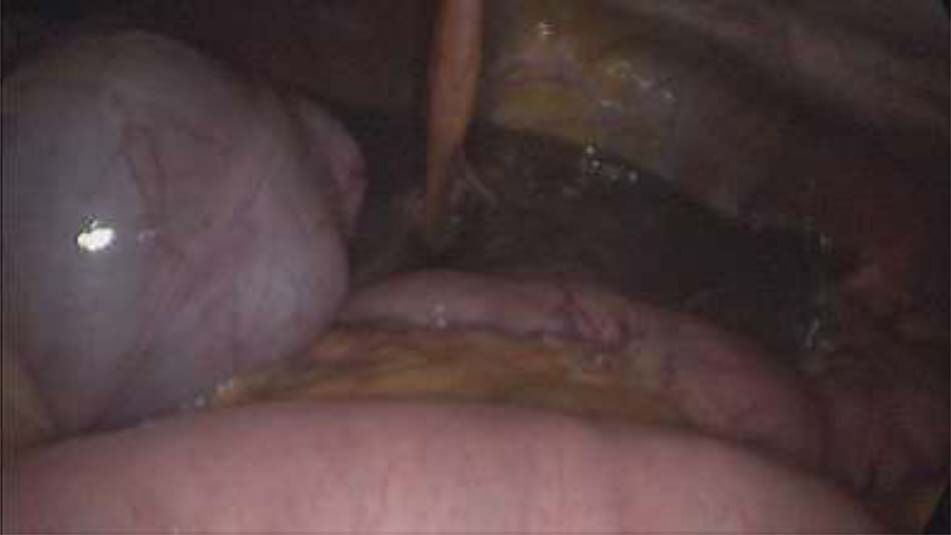
Showing floppy dilated cecum ileum and ascending colon twisted around the liver and mesentery.

**Figure 7:**
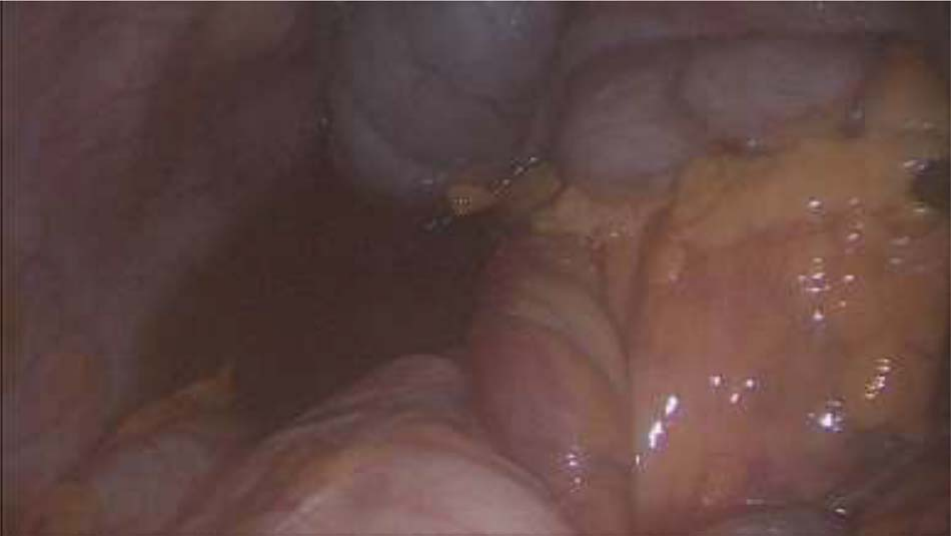
Turbid free fluid in the right para-colic gutter.

Emergency diagnostic laparoscopy was performed and findings were: 180 degree volvulus of terminal ileum, cecum and ascending colon overlapped each other and overriding the right loop of the liver, the lateral attachment of the right colon was long dilated and floppy (Figs 5 and 6**)**. The dudeno-jejenal flexure was malrotated and lying in left upper quadrant just below to splenic flexure. Three congenital bands were found tilted around the terminal ileum, cecum and base of the appendix. Turbid free fluids with no evidence of infection also noted in the right upper quadrant and right para-colic gutter (Fig. 7). Otherwise, bowels were found healthy and viable. A healthy retrocecal and subserosal appendix was also noted intraoperatively. We converted to open Laparotomy and proceeded with right hemicolectomy due to complex intraoperative findings. The postoperative period was uneventful, and patient was discharged at home on postoperative Day 7.

## DISCUSSION

To the best of our knowledge, caecal volvulus associated with this complex small bowel malrotation and congenital peritoneal bands in a previously fit and well middle-aged female has never been reported in literature. Approximately, in ~85% cases the detection of intestinal malrotation usually takes place within the first 2 weeks after birth [10]. Midgut malrotation is an anomaly of fetal intestinal rotation that usually presents in the first month of life. It is rare for malrotation to present in adulthood [11].

This condition often goes undetectable, especially in a patient with no past medical history, and hence the clinical diagnosis at the time of presentation is of great difficultly [6]. Therefore, in patients with recurrent intermittent or persistent abdominal pain, caecal volvulus should be considered. Also, the awareness about such condition is essential as can lead to earlier identification and treatment of this potentially lethal gastrointestinal tract problem and safe patient’s life.

Although an abdominal X-ray can be helpful in terms of diagnosis but not in all conditions, CT scan is an investigation of choice for such cases of caecal volvulus to make solid diagnosis and operate based on that evidence. In our patient the caecum, ileum and ascending colon were found entrapped and twisted at right hepato-diaphragmatic space resulting in an unusual Chilaiditi syndrome; hence a chest X-ray may give false impression of bowel perforation. Therefore, clinical correlation is always mandatory in order to consider different management options as in such cases.

Coexistence of congenital intestinal malrotation in adults precipitates more risks of developing other types of colonic volvulus [12]. Therefore, postoperative counseling and communication with patients and families about possible future risks of volvulus and obstruction can steer to a limpid risk reduction and avoidance of late presentation and hence improves the management outcome.

Surgery is the preferred method of treatment for caecal volvulus [13]. Right hemicolectomy can be also applied to caecal volvulus even with no bowel necrosis in order to prohibit the future risk of recurrence, mortality and developing other types of colonic volvulus. Conservative management is not recommended because of the high risk of ischemia, and surgical detorsion alone carries a high rate of recurrence (20%–75%) [14]. Minimal invasive procedures such as caecopexy or endoscopic detwisting of caecal volvulus with viable bowel are of no proven benefits as they can risk both the prognosis and outcome.

In conclusion, we describe a rare and complex case of caecal volvulus associated with complex bowel loops malrotation of ileum, cecum and ascending colon in a single middle-aged patient with intermittent bowel symptoms has never been reported in literature. Therefore, in patients with recurrent intermittent or persistent abdominal pain, caecal volvulus should be considered. Authors focus on raising awareness among the doctors covering emergency department about the importance of considering caecal volvulus as a differential diagnosis of acute abdomen during their initial assessment of patients with intermittent bowel symptoms in order to avoid any therapeutic delay that can lead to serious intra-abdominal complications. This awareness may lead to earlier identification and treatment of this potentially lethal gastrointestinal tract problem.

## CONFLICT OF INTEREST STATEMENT

None declared.

## FUNDING

No funding was received for this study.

## INFORMED CONSENT

Informed consent has been obtained from the patient to use this data for publication.
