# Corrigendum to: Caecal volvulus associated with complex small bowel malrotation and congenital bands: A case report and literature review

**DOI:** 10.1093/jscr/rjab214

**Published:** 2021-05-19

**Authors:** Hussam S Khougali, Yousif Hamad, Ahmed Abdelhamid, Valerio DiNicola

**Affiliations:** Department of General Surgery, Worthing Hospital, Worthing, UK

When this paper was originally published, Valerio DiNicola was originally missing as the fourth author. This has now been corrected online.

